# Impact of Highly Effective CFTR Modulator Therapy on Physical Activity, Sleep, and Sinonasal Symptoms in Preschool Children with Cystic Fibrosis: A Prospective Single-Center Pilot Study

**DOI:** 10.3390/arm94030036

**Published:** 2026-06-05

**Authors:** Stella Schellhorn, Hanna Schmidt, Ales Janda, Doris Gülke, Monika Toth, Dorit Fabricius, Sebastian F. N. Bode

**Affiliations:** Department of Pediatrics and Adolescent Medicin, Ulm University Medical Center, Ulm University, Eythstrasse 24, 89075 Ulm, Germany

**Keywords:** cystic fibrosis, preschool children, CFTR-modulator, wearables, actigraphy, activity, sleep, sinonasal symptoms

## Abstract

**Highlights:**

**What are the main findings?**

- ETI therapy in 2–6 year olds led to a temporary increase in physical activity measured by actigraphs (step count and moderate-to-vigorous activity at 3 and 6 months), which returned to near-baseline levels after 12 months, while parental perception of physical performance improved sustainably.
- Sleep parameters measured by actigraphs and sinonasal symptoms proxy-rated by parents remained stable.

**What are the implications of the main findings?**

- The observed improvement in physical activity may be influenced by external confounders such as seasonality, highlighting that treatment effects should be interpreted cautiously in small, uncontrolled cohorts.
- Sleep parameters as measured by actigraphs remained stable in small children supporting the safety of ETI in preschool children, but larger, controlled longitudinal studies are needed to confirm these findings.

**Abstract:**

**Background**: Highly effective CFTR modulation with Elexacaftor/Tezacaftor/Ivacaftor (ETI) markedly improves clinical outcomes in people with cystic fibrosis (CF). Data on its effects on physical activity, sleep, sinonasal symptoms, and parent-perceived outcomes in preschool-aged children are limited. **Methods**: In this prospective, observational, single-center cohort study, ten children with cystic fibrosis (aged 2–6 years) and at least one *CFTR* variant eligible for ETI were included. Data were collected using wrist-worn Garmin vívofit Junior 2 activity trackers and standardized questionnaires one month before ETI initiation and at 1, 3, 6, and 12 months after start of ETI. Outcomes included step count, minutes of moderate-to-vigorous physical activity, sleep parameters, sinonasal symptoms, and parental perceptions. **Results**: ETI was well tolerated. Sweat chloride levels decreased significantly. Physical activity improved at 3 and 6 months (step count and active minutes/day; *p* < 0.05) but declined to near-baseline levels at 12 months. Parental assessments of physical and sporting performance showed sustained improvement. Sleep duration remained stable, with no changes in deep or light sleep phases or nighttime awakenings. Sinonasal symptoms remained low. **Discussion & Conclusions**: Preliminary findings of this exploratory pilot study show that improvement in physical activity after three and six months of ETI therapy might be attributable to seasonality, as therapy was started in winter months. No changes in sleep duration or sleep patterns are reassuring in this small cohort of young children with CF. ETI therapy was safe and well tolerated. Parental appraisal of their children’s physical performance improved after start of ETI. Longitudinal, controlled studies involving larger cohorts are required to validate these findings and to account for potential confounding factors, such as age-dependent changes and individual and environmental factors such as seasonal variation.

## 1. Background

Cystic fibrosis (CF) represents the most prevalent life-limiting autosomal recessive disorder among individuals of Caucasian descent. The disease results from pathogenic variants in the *CFTR* gene, which encodes an epithelial ion channel mediating chloride and bicarbonate transport. *CFTR* dysfunction leads to viscous secretions in the respiratory tract, pancreas, gastrointestinal tract, and other organs, causing chronic respiratory infections, pancreatic insufficiency, and malnutrition [1,2]. In recent years, CFTR modulator therapies have revolutionized treatment by targeting the underlying molecular defect and improving CFTR protein function, leading to markedly increased longitudinal survival in people with CF (pwCF) who carry CFTR-modulator-responsive *CFTR* variants [3,4]. With the approval of Elexa-/Teza-/Ivacaftor (ETI) for children with CF and at least one *CFTR* residual function variant from the age of two years, for the first time, even toddlers with CF can receive highly effective CFTR modulator therapy [5].

Children with CF historically have been less physically active than their healthy peers [6]. Both pulmonary limitation and time-consuming treatment of CF, requiring multiple daily inhalation sessions, physiotherapy, and medication contributed. Physical activity and exercise are important components of the interdisciplinary treatment of CF [7,8,9]. Although large randomized controlled trials are still lacking and meta-analyses have yielded inconsistent results [9], there is substantial evidence suggesting a positive impact of physical activity and exercise on the course of the disease. Regular physical activity has been associated with a slower decline—or even improvement—in lung function parameters [10], improved mucus clearance [7], and strengthening of the respiratory muscles [11], as well as indications of prolonged survival [12] and enhanced quality of life [13]. Even with the availability of highly effective CFTR modulator therapies, these treatment approaches not only support mucus clearance and disease stabilization but also offer psychosocial benefits by emphasizing personal resources rather than deficits and illness.

PwCF have been reported to suffer from relevant sleep disturbances including frequent awakenings, snoring, day-time sleepiness, and hypoxemia in advanced CF before the advent of highly effective CFTR-modulator therapy [6,14,15,16]. Some of these symptoms might be attributable to obstructive sleep apnea [16], which might be partially triggered by upper airway disease as mucosal swelling and nasal polyps in PwCF. Therapy with ETI has been shown to improve sinonasal symptoms in adults and in children with CF older than 6 years [17,18]. New onset of sleep disturbances after start of ETI in children as well as adolescents has been discussed controversially [19,20], and in a small sample of young adults self-perceived sleep quality even improved after ETI [21].

There is a research gap on whether both sleep and sinonasal symptoms contribute to change in quality of life after initiation of ETI. This prospective single-center pilot study objectively assesses the physical activity and sleep patterns using an activity/sleep tracker in children with CF aged 2–6 years and at least one *CFTR* variant eligible for ETI before and up to one year after initiation of therapy with ETI. These data are complemented by standardized questionnaires on activity and sinonasal symptoms filled in by parents or caregivers.

## 2. Methods

**Figure 1:**
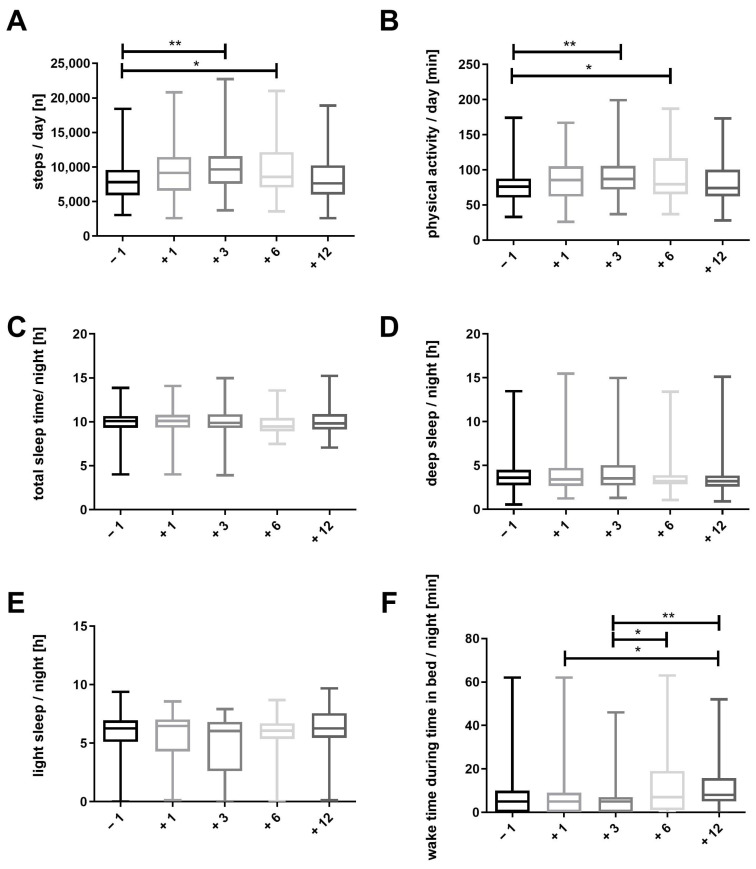
(**A**) steps per day, (**B**) minutes of moderate to vigorous physical activity. In (**A**,**B**), the grey area represents spring and summer months during the study period. (**C**) total sleep time in hours per night, (**D**) deep sleep time in hours per night, (**E**) light sleep time in hours per night, and (**F**) wake time during time in bed per night in minutes before (−1) and 1 month (+1), 3 months (+3), 6 months (+6), and 12 months (+12) after start of ETI. *: *p* < 0.05, ** *p* < 0.001.

This study was designed per STROBE guidelines as a prospective, observational, single-center cohort pilot study. All families of children with genetically confirmed CF aged 2–6 years who had at least one *CFTR* variant eligible for ETI and therefore eligible for ETI therapy were offered participation in the study. Both verbal and written information about the study was provided, and a signed informed consent form was obtained from all parents of participants prior to study initiation. Given the age of the enrolled children, consent was signed by their parents or legal guardians. The children were informed about the study in an age-appropriate manner using simple language and were asked to provide verbal assent. The recruitment period was from 1 December 2023 until 31 March 2024. All consenting families were provided with questionnaires to be filled in by paper and pencil and an activity tracker one month before the start of ETI therapy, as well as one month, three months, six months, and 12 months after the start of ETI (Figure 1).

Families and children were instructed to wear the activity tracker on their wrist or ankle day and night for seven consecutive days at each assessment point. Prior to distribution, the activity tracker was thoroughly cleaned and disinfected. The start and end of time of wearing the activity tracker was noted by the parents, and the trackers were sent back via postal services. The devices were thoroughly cleaned and desinfected again. Each participant served as their own control before and after the start of ETI therapy.

Data collection was conducted using an adapted version of the “Questionnaire for Measuring Physical Activity and Sports Participation” (BSA Questionnaire), the “Scales on Sport-Related Situational Barriers and Barrier Management” [22,23], and the Sino-Nasal Outcome Test-22 (SNOT-22) [24] evaluating parents’ perceived changes in the child’s energy, endurance, physical activity, sleep quality, and sinonasal symptoms. SNOT-22 is not validated in children < 6 years but was used as it was proxy-rated by the parents. The SNOT-22 was chosen as it is routinely administered to all patients with cystic fibrosis and all parents of children with CF during outpatient visits at our institution. To ensure consistency with standard clinical practice and to avoid additional respondent burden, other validated proxy-reported instruments, such as the SN-5, were not included. Extensive research regarding an optimal activity tracker for children 2–6 years old was done prior to the start of the study. There was no commercially available device that aims specifically at this age group. We selected the Garmin vívofit Junior 2 (Garmin Ltd., Olathe, KS, USA), as this device stores data locally on the device. No real-time tracking of any participants is possible with this device. Additionally, the device is small and lightweight and has minimal screen distraction. After returning the tracker, data on minutes of moderate-to-vigorous physical activity (in minutes per day), steps taken (steps per day), and sleep (categorized into total sleep time, deep and light sleep phases, nocturnal awake phases) were transferred via Bluetooth to a secured mobile device. After transfer, the data were deleted from both the app on the mobile device and the activity tracker. All data from the questionnaires and activity trackers were anonymized and then compiled in a local Microsoft Excel (Microsoft Corporation, Richmond, VA, USA) file on a secured local server.

### 2.1. Data Analysis and Statistics

Means, standard deviations, and confidence intervals for each parameter were calculated for every time point. Data were supplemented from the local hospital information system regarding sweat chloride, pulmonary function tests, biometric data such as weight and height, and laboratory data, especially liver function tests (performed at the same time points as mentioned above). Data were analyzed using Microsoft Excel and GraphPad Prism Version 6.07 (Prism, La Jolla, CA, USA). Categorical variables are presented as frequencies, while descriptive statistics including mean, standard deviation, median, minimum, maximum, and quartiles are reported as appropriate. Longitudinal data were analyzed using repeated-measures analysis of variance (ANOVA) or the Kruskal–Wallis test with Dunn’s post hoc multiple-comparison procedure, where applicable. Statistical significance was defined as a two-sided α level of <0.05. Free-text comments were analyzed according to quantitative content analysis [25].

### 2.2. Ethics

Ethical approval was obtained from the local University Ethics Committee (Reference No. 289/23).

## 3. Results

Out of 14 families with children 2–6 years old at our center, 12 families consented to participate in the study. Two families were unable to use the step counters and complete the questionnaires within the required study periods. Ten children were included in the study. Eight children had F508del homozygous *CFTR* variants and two had f508del heterozygous variants. N = 5 girls and n = 5 boys were included in the study, with an average age of m = 4.07 years (standard deviation (SD) ± 1.27, confidence interval (CI) 3.16–4.99). All participants started ETI therapy between December 2023 and February 2024. No therapy-related adverse events were observed. No changes in liver function parameters were found. No pulmonary exacerbations were noted in the study period. The children’s weight gain followed their individual percentile trajectories. Sweat chloride decreased from 94.6 (±11.7) mmol/L before to 31.4 (±10.3) mmol/L after three months of ETI therapy (*p* ≤ 0.001). Pulmonary function tests were performed in all participants > 4 years, but due to age-related limited cooperation, valid results could not be obtained in the majority of cases, and further analysis was therefore omitted.

### 3.1. Physical Activity and Sleep

The average number of daily steps increased significantly three months (*p* < 0.01) and six months (*p* < 0.05) after start of ETI but returned to baseline levels at 12 months (Figure 1A and Table 1).

Minutes per day of moderate-to-vigorous physical activity rose significantly after three months (*p* < 0.01) and six months (*p* < 0.05) after start of CFTR modulator therapy. After 12 months it was higher than before ETI, but differences were not significant (Table 1 and Figure 1B).

Average total sleep time did not change significantly during the study (Figure 1C and Table 2). There were no significant changes in the proportions of deep and light sleep (Table 2 and Figure 1D,E). Regarding wake time during time in bed there was an increase over time—but no significant changes compared to prior initiation of ETI therapy (Table 2 and Figure 1F).

### 3.2. Sinonasal Symptoms

**Figure 2:**
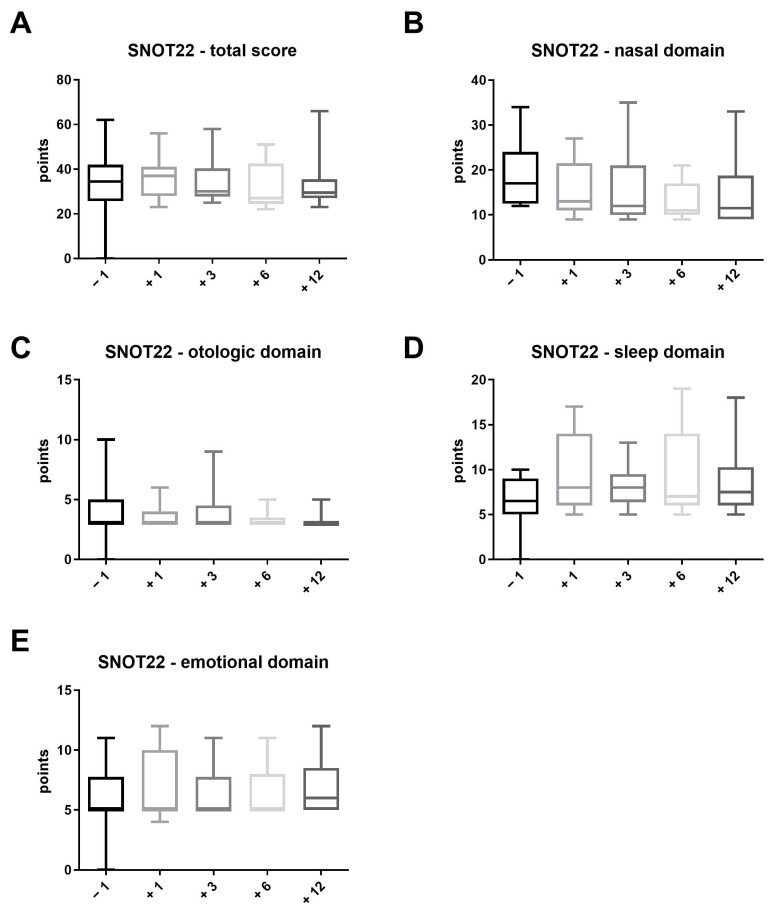
(**A**) SNOT-22 total score, (**B**) SNOT-22 nasal domain, (**C**) SNOT-22 otologic domain, (**D**) SNOT-22 sleep domain and (**E**) SNOT-22 emotional domain before (−1) and 1 month (+1), 3 months (+3), 6 months (+6), and 12 months (+12) after start of ETI. Mean, standard deviation, and individual values are displayed. The maximum achievable score is shown on each respective y-axis; higher scores indicate more severe symptoms.

No significant changes were observed over the course of the study in the total SNOT-22 score or in any of the subdomain scores, including nasal, otologic, sleep-related, and emotional domains (Figure 2A–E and Table 3).

### 3.3. Subjective Findings

**Figure 3:**
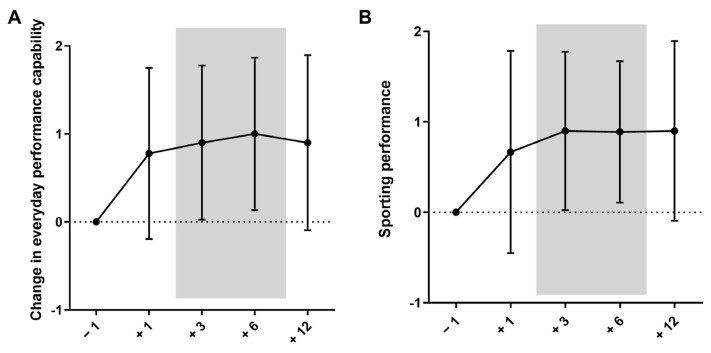
Subjective parental assessments of (**A**) everyday performance capacity and (**B**) sporting performance before (−1) and 1 month (+1), 3 months (+3), 6 months (+6), and 12 months (+12) after start of ETI. The grey area represents spring and summer months during the study period. Means, minima, and maxima are displayed. “0” = no change; “1” = slight improvement; “2” = marked improvement.

One third of caregivers reported a marked improvement both in their children’s overall physical fitness and sporting performance after six months of ETI therapy, with another third reporting moderate improvement. No parents reported a decline, and changes were not significant (Figure 3A,B). Over the 12-month observation period, 70% of children regularly took part in physical activity. On average, children engaged in 76.4 (±59.1) minutes of sports per week in the four weeks prior to starting Elexacaftor/Tezacaftor/Ivacaftor (ETI) therapy. After one month of ETI, this dropped to 52.3 (±65.1) minutes per week, followed by an increase to 140.5 (±179.6) minutes per week after three months. At six months, the average was 107.5 (±146.0) minutes per week, declining again to 60 (±73.8) minutes per week at 12 months. These fluctuations were not statistically significant. No changes were observed in everyday walking, bicycle use, and stairs climbed according to parents’ estimation. Outdoor play occurred on 11.3 days/month before ETI therapy and peaked at 20.4 days at three months. By 12 months, this dropped to 8.7 days.

Regarding the nature of activities, children preferred doing sports in pairs or groups. Gymnastics, cycling, and trampoline jumping were the most popular activities. When asked about potentially interesting physical activities, children showed a consistent interest in gymnastics, trampoline jumping, cycling, and—seasonally—swimming. The most significant barriers to engaging in physical activity included illness, limited time due to therapy commitments, the high cost of sports programs, and unfavorable weather, as well as the comfort of home and the appeal of television entertainment.

## 4. Discussion

This is one of the first pilot studies that objectively measures physical activity and sleep with a wearable device tracking minutes of moderate-to-intense physical activity and sleep patterns in a small cohort of children aged 2–6 years with CF who started highly effective CFTR-modulator therapy. All results of this exploratory pilot study should be interpreted with caution due to the small number of participants and cannot be generalized. ETI was well tolerated, no adverse events were reported, and sweat chloride levels fell similarly as reported before [5], hinting at both efficacy and adherence to the therapy. One other study using activity trackers in children with CF to monitor sleep has been published—but no reports on use of ETI or other modulator therapies have been included by the authors [26].

Both daily step counts and minutes of moderate-to-vigorous physical activity, as assessed by activity trackers, increased following initiation of ETI therapy—most notably at three and six months—but largely returned to baseline levels after one year of treatment. Parental assessment of their children’s everyday and sporting performances showed sustained improvement throughout the study period. Activity trackers combined with personalized feedback to encourage physical activity have been used in adults with CF [27]. There is some evidence that physical activity in adults, measured by an activity tracker, increases after initiation of ETI [28]. Our results show only temporary improvement. Taking into account that ETI was started between December and May and most improvement was seen after three and six months, which translates to spring and summer months in Europe, the improvement in physical activity might be attributable to warmer temperatures and more possibilities of physical activity outside. Seasonal patterns have been previously described as a major factor influencing outdoor activity levels in pediatric populations [29]. Additionally young children show age-dependent changes with increasing age either with improved coordination, balance, and endurance but also a natural decline in physical activity even in childhood up to school age [30]. Due to the small cohort and lack of control group, no statistical adjustment for weather, outdoor activities, or daycare/school attendance could be carried out.

The World Health Organization (WHO) recommends 180 min of total physical activity per day for children under 5 years, including 60 min of moderate- to vigorous-intensity and vigorous-intensity activities at least three days per week [31]. Participants in this study exceeded these recommendations on moderate-to-vigorous activity throughout the study period as measured by the activity trackers, but we found a wide range of physical activity among the different participants, as has been shown previously [30]. Low-intensity physical activity was hard to track with the activity tracker used, as this activity was not reported by the device, and it is not published how the algorithm within the tracker differentiates between levels of activity. The WHO recommendation on at least 3 days per week with vigorous activities is also harder to track with the design of this study. Most participants at least performed some guided activities such as soccer, kids’ gymnastics, swimming, etc., and a marked increase in parental satisfaction with their children’s physical abilities was observed, but all those data are proxy-reported by parents with a questionnaire.

In this cohort, no significant changes in sleep duration with a sleep duration around 10 h per night were found. Light and deep sleep phases as measured by accelerometers were found unchanged after initiation of ETI. There was a slight increase in awake times over the course of the study. Healthy children aged 2–6 years normally sleep around 9–10 h a night. Rapid-eye-movement (REM) sleep in children in general gradually decreases to about 25% of total sleep time. Light sleep phases measured by polysomnography account for around 40–50% of sleep time and deep sleep phases for 30–35% [32]. The setup of this study cannot differentiate between REM and non-REM sleep. Sleep duration and sleep architecture regarding deep sleep and light sleep in the cohort reported here are similar to that of healthy children. The cohort of this study is very young so that previously reported sleep problems in CF [14,15,16,26] might not have manifested yet. Neither sleep duration nor sleep architecture as measured by accelerometer in the activity tracker changed after ETI, and parental reports in the SNOT-22 sleep domain showed no change. There was a non-significant increase in wake time during total sleep time, which might represent awakenings. These changes occurred during the study period where all participants already received ETI—and no changes compared to initiation of therapy were found. Polysomnographic studies are needed to evaluate nighttime awakenings. New onset of sleep disturbances measured by questionnaires after start of ETI in children and adolescents have been reported [19,20]. Even though the study presented here can only give an indication of sleep quality and cannot be compared with the gold standard of polysomnography, it is reassuring that in a real-world setting no significant changes in sleep patterns could be identified after start of ETI compared to baseline.

Sinonasal symptoms in the cohort reported here remained unchanged over the study period. Symptom scores were initially low, with a mean total SNOT-22 score of 33.3 points (out of a maximum of 110), and similarly low scores were observed in the nasal, otologic, sleep, and emotional subdomains, which remained stable throughout the study. Improvement in sinonasal symptoms and quality of life in children 6–12 years after start of ETI have been reported [33], as have improvements measured by MRI [34]. This discrepancy between published data and the results presented here might be due to the younger age of the cohort, with fewer CF-related sinonasal problems and more infection-associated sinonasal symptoms typical at the 2–6-years-old age group. The present study design does not allow for a clear distinction between these two potential causes.

## 5. Limitations and Strengths

The primary limitation of this pilot study is the small sample size, comprising only 10 children. Additionally, no control group could be included in the study, as all parents of children with CF in the respective age group at our institution chose to start CFTR modulator treatment. Although wearable devices provided objective measurements of physical activity and sleep, their accuracy in very young children may be limited due to wearing compliance, frequent removal, improper placement due to small limb size, and reduced algorithm accuracy for short, irregular movement patterns typical of preschool children. In addition, energy expenditure and step-count algorithms are often extrapolated from adult-based models, which may not accurately reflect physiological and behavioral patterns in this age group. Consequently, while wearables can provide useful relative trends, absolute values should be interpreted with caution. Also, the duration of one week of wearing the activity tracker at all time points might be too short and influenced by short changes in weather or acute infections. No clear distinction of sleep phases was possible with the device, and results should be interpreted with caution. Furthermore, activity and sleep patterns are influenced by environmental and behavioral factors, including weather, infections, daycare/school attendance, and parental routines, which were not systematically controlled. Seasonal variation, particularly the winter-to-summer transition and the return to winter, likely influenced physical activity trends. Young children also show improvement in both endurance and skills, which might contribute to a more positive perception of parents. The absence of a control group without ETI therapy limits the ability to attribute observed changes solely to pharmacological treatment. Children acted as their own control before and after start of ETI, and no children with CF without at least one *CFTR* variant eligible for ETI in the same age group were available at our center to be included in the study as controls. Withholding ETI treatment in children eligible for the therapy would be unethical. Strengths of the study are the real-world design with combination of activity trackers providing objective data and subjective parental assessments by questionnaires. The relatively long follow-up period of 12 months and repetitive assessments in the young age group can be considered positively, but due to the small sample size no generalizable conclusions should be drawn from the data presented here.

## 6. Conclusions

ETI was well tolerated in a small cohort of children with CF aged 2–6 years. Sweat chloride showed a marked decrease. Objectively measured moderate-to-vigorous physical activity did improve in the spring and summer months following initiation of ETI but returned to baseline in winter months. Parental satisfaction with positive development of their children’s physical activity and sporting performance was high after start of ETI. No changes in sleep duration or sleep patterns, as measurable with a wearable activity tracker, were observed. Sinonasal symptoms, measured with the SNOT-22 questionnaire, remained at low levels after initiation of ETI, hinting that possibly not only cystic fibrosis plays a role in sinonasal symptoms in preschool children with CF. The small sample size prohibits generalization of the results to the general CF population. Confounding factors such as seasonality, weather, infections, daycare/school attendance, outdoor activities, etc. should be taken into account when designing larger follow-up studies.

## Figures and Tables

**Table 1:** Steps per day and moderate-to-vigorous physical activity in minutes before and after initiation of ETI. Means, standard deviations, and 95% confidence intervals are reported.

Activity	−1 Month	+1 Month	+3 Months	+6 Months	+12 Months
**Steps/day**	7965 ± 2791, 7348–8582	9087 ± 3571, 8236–9938	9836 ± 3471, 9032–10,640	9762 ± 3607, 8959–10,564	8502 ± 3437, 7756–9248
**Activity [min]**	76.0 ± 28.5, 76.6–90.2	83.4 ± 28.5, 76.6–90.2	90.9 ± 29.8, 84.0–97.8	91.5 ± 32.6, 84.3–98.8	81 ± 29.3, 75.1–87.8

**Table 2:** Sleep times during the study. Means, standard deviations, and 95% confidence intervals are reported.

Sleep Pattern	−1 Month	+1 Month	+3 Months	+6 Months	+12 Months
**Total sleep/day [h]**	10.1 ± 1.6 9.8–10.5	10.1 ± 1.8 9.7–10.5	10.1 ± 1.9 9.7–10.5	9.8 ± 1.4 9.5–10.1	9.9 ± 1.2 9.7–10.2
**Deep sleep/day [h]**	4.7 ± 3.4 3.9–5.5	5.0 ± 4.0 4.1–5.9	5.2 ± 3.8 4.3–6.1	4.2 ± 3.1 3.5–4.9	3.5 ± 1.7 3.1–3.8
**Light sleep/day [h]**	5.5 ± 2.4 4.9–6.0	5.2 ± 2.7 4.6–5.8	4.9 ± 2.7 4.2–5.5	5.6 ± 2.1 5.1–6.1	6.5 ± 1.6 6.2–6.9
**wake time during time in bed/day [min]**	9.4 ± 14.1 6.4–12.4	7.9 ± 11.6 5.3–10.5	7.5 ± 11.5 5.0–10.1	12.4 ± 13.9 9.1–15.6	11.0 ± 10.5 8.7–13.3

**Table 3:** SNOT-22 score including subdomains during the study. Means, standard deviations, and 95% confidence intervals are reported.

SNOT-22	−1 Month	+1 Month	+3 Months	+6 Months	+12 Months
**Total score [points]**	33.3 ± 15.7 22.1–44.5	36.2 ± 9.8 28.7–43.8	34.5 ± 10.6 26.9–42.0	32.33 ± 10.5 24.3–40.4	33.3 ± 12.5 24.4–42.2
**Nasal domain [points]**	18.8 ± 7.3 13.2–24.4	15.4 ± 6.3 10.6–20.3	15.8 ± 8.7 9.6–22.0	13.0 ± 4.5 9.56–16.4	14.6 ± 7.6 9.1–20.1
**Otologic domain [points]**	4.1 ± 2.8 2.1–6.1	3.6 ± 1.1 2.7–4.4	4.0 ± 2.0 2.6–5.4	3.4 ± 0.7 2.8–3.9	3.2 ± 0.6 2.7–3.6
**Sleep domain [points]**	6.5 ± 3.1 4.3–8.6	9.7 ± 4.3 6.4–12.9	8.3 ± 2.3 6.6–9.9	9.7 ± 4.9 5.8–13.5	8.6 ± 3.8 5.9–11.3
**Emotional domain [points]**	5.8 ± 3.0 3.6–7.9	7.0 ± 2.9 4.7–9.2	6.4 ± 2.3 4.8–8.0	6.3 ± 2.2 4.6–8.0	6.9 ± 2.4 5.2–8.6

## Data Availability

Data are available from the corresponding author upon reasonable request.
